# Supplementary material for: iCanClean Removes Motion, Muscle, Eye, and Line-Noise Artifacts from Phantom EEG
Source: Sensors (Basel). 2023 Oct 1;23(19):8214. doi: 10.3390/s23198214 (PMC10574843; doi:10.3390/s23198214)
Supplement: Supplementary file 1 [file sensors-23-08214-s001.zip › File S1.pdf]

Mathematics of iCanClean

Mathematical detail of the iCanClean algorithm is provided below, with key equations numbered and written in MATLAB syntax using built-in MATLAB functions. **Variables** are bolded. *Functions* are italicized. A list of variables is provided in Table 1, along with their description and dimensions.

Table S1. List of variables.

Symbol	Description	Dimensions
**A**	Unmixing matrix (data channels)	$\mathbf{N}_{\mathbf{Data}} \times \mathbf{N}_{\mathbf{Comp}}$
**B**	Unmixing matrix (noise channels)	$\mathbf{N}_{\mathbf{Noise}} \times \mathbf{N}_{\mathbf{Comp}}$
**BadCompActivity**	Noise components selected for removal	$\mathbf{T} \times \mathbf{N}_{\mathbf{Bad}}$
**BadCompList**	Indexed list of bad components	$\mathbf{N}_{\mathbf{Bad}} \times 1$
$\mathbf{N}_{\mathbf{Bad}}$	Number of noise components selected for removal	Scalar
$\mathbf{N}_{\mathbf{Comp}}$	Number of candidate noise components	Scalar
$\mathbf{N}_{\mathbf{Data}}$	Number of data channels	Scalar
$\mathbf{N}_{\mathbf{Noise}}$	Number of noise channels	Scalar
**ProjectionMatrix**	Relates noise component space to data channel space	$\mathbf{N}_{\mathbf{Bad}} \times \mathbf{N}_{\mathbf{Data}}$
**ProjectedNoise**	Bad noise components projected onto the data channels	$\mathbf{T} \times \mathbf{N}_{\mathbf{Data}}$
**R**	Correlation between each ($\mathbf{U}_i$, $\mathbf{V}_i$) pair i = [1, 2, …, $\mathbf{N}_{\mathbf{Comp}}$]	$\mathbf{N}_{\mathbf{Comp}} \times 1$
**T**	Number of time points (samples)	Scalar
**Thresh**	User defined threshold for identifying bad components	Scalar
**U**	Candidate noise components (calculated from data channels)	$\mathbf{T} \times \mathbf{N}_{\mathbf{Comp}}$
**V**	Candidate noise components (calculated from noise channels)	$\mathbf{T} \times \mathbf{N}_{\mathbf{Comp}}$
**X**	Corrupted data recordings	$\mathbf{T} \times \mathbf{N}_{\mathbf{Data}}$
$\mathbf{X}_{\mathbf{Clean}}$	Cleaned data	$\mathbf{T} \times \mathbf{N}_{\mathbf{Data}}$
$\mathbf{X}_{\mathbf{MC}}$	Mean centered data	$\mathbf{T} \times \mathbf{N}_{\mathbf{Data}}$
**Y**	Reference (or pseudo reference) noise recordings	$\mathbf{T} \times \mathbf{N}_{\mathbf{Noise}}$
$\mathbf{Y}_{\mathbf{MC}}$	Mean centered noise	$\mathbf{T} \times \mathbf{N}_{\mathbf{Noise}}$

Definitions

Let **X** be the corrupted data recordings the user wishes to clean with dimensions $\mathbf{T} \times \mathbf{N}_{\mathbf{Data}}$, where **T** is the number of time points (or samples) and $\mathbf{N}_{\mathbf{Data}}$ is the number of data channels. Similarly, let **Y** be the reference (or pseudo reference) noise recordings with dimensions $\mathbf{T} \times \mathbf{N}_{\mathbf{Noise}}$, where $\mathbf{N}_{\mathbf{Noise}}$ is the number of reference noise channels.

Step 1

Given corrupted data to clean (**X**) and reference (or pseudo reference) noise recordings (**Y**), canonical correlation analysis is used to identify latent sources of noise (i.e., candidate noise components) in common to both **X** and **Y**.

[**A** , **B** , **R** , **U** , **V**] = *canoncorr*(**X** , **Y**)	(1)

A is an unmixing matrix that converts corrupted data recordings to candidate noise components as $\mathbf{U} = \mathbf{X}_{\mathbf{MC}}{*}\mathbf{A}$, where $\mathbf{X}_{\mathbf{MC}}$ is the mean centered data. Similarly, **B** is an unmixing matrix that converts reference noise recordings to a second set of candidate noise components as $\mathbf{V} = \mathbf{Y}_{\mathbf{MC}}{*}\mathbf{B}$. Finally, **R** is a vector which contains the correlation between each unique ($\mathbf{U}_i$, $\mathbf{V}_i$) pair, where $\mathbf{U}_i$ and $\mathbf{V}_i$ are the ith columns of **U** and **V**, respectively. The number of candidate noise components identified, $\mathbf{N}_{\mathbf{Comp}}$, depends on the rank of the data. Specifically, $\mathbf{N}_{\mathbf{Comp}} = min(\, rank(\mathbf{X}) , rank(\mathbf{Y})\,)$. Therefore, $\mathbf{N}_{\mathbf{Comp}} \leq min(\, \mathbf{N}_{\mathbf{Data}} , \mathbf{N}_{\mathbf{Noise}})$.

Commentary: With the iCanClean approach, we use corrupted data recordings as one set of inputs to canonical correlation analysis (CCA) and reference noise recordings as the second set of inputs. CCA seeks to find the subspaces of two datasets which are maximally correlated with each other. Because the corrupted data recordings and reference noise recordings both contain noise, CCA will identify hidden noise components that are common to both datasets. Canonical correlation analysis returns candidate components in ranked order. Thus, the noise component pair with the strongest relationship (i.e., largest $\mathbf{R}^2$ correlation) appears first ($\mathbf{U}_1$, $\mathbf{V}_1$). The next noise component pair ($\mathbf{U}_2$, $\mathbf{V}_2$) has the second largest $\mathbf{R}^2$ correlation, and so forth. Note that the second component pair is solved with the constraint that it be uncorrelated to the first component pair, and similarly the third component pair is constrained to be uncorrelated to the first two component pairs, and so on. Specifically, $\mathbf{U}_i$-$\mathbf{U}_j$ are uncorrelated, $\mathbf{V}_i$-$\mathbf{V}_j$ are uncorrelated, $\mathbf{U}_i$-$\mathbf{V}_j$ uncorrelated, and $\mathbf{V}_i$ -$\mathbf{U}_j$ are uncorrelated, wherever $i \neq j$. The noise components identified by canonical correlation do not depend on how strongly the noise sources project onto the data channels or noise channels. Thus, both large and small noise sources are identified. In a subsequent step, candidate noise components will be appropriately scaled to match the channels being cleaned.

Step 2

A bad subset of components is identified. Here we use a basic thresholding technique where all components with a squared correlation value ≥ a user-defined threshold are rejected. The user has a choice of whether to select **U** or **V**, or a combination of **U** and **V** as their noise components. The best choice may vary by each specific application. In (3), we assume the user wishes to use mixtures (or subspaces) of the data channels to clean the data channels themselves (i.e., use **U** to clean **X**). In (3b) and (3c), we give alternative options which include mixtures (subspaces) of the noise channels.

**BadCompList** = *find*(**R**.^2 >= **Thresh**)	(2)
**BadCompActivity** = **U**(: , **BadCompList**)	(3)

**BadCompActivity** = **V**(: , **BadCompList**)	(3b)
**BadCompActivity** = **U**(: , **BadCompList**)/2 + **V**(: , **BadCompList**)/2	(3c)

Commentary: When identifying a bad subset of components, alternative methods could be employed, as opposed to the simple thresholding technique used in equation 2. For example, a fast Fourier transform (FFT) could be calculated on the components (U,V) to identify components based on their power spectral profiles. Similarly, in lieu of a single, common thresholding value in equation 2, multiple R^2 thresholds could be determined on a per-component-basis. To aid in selecting the thresholding value(s), data of interest could be randomly resampled or a separate set of calibration data could be referenced. We generally suggest using **U** to define the bad components (rather than **V**). This is because the intent is to clean the corrupted data channels (**X**). Artifacts, specifically as they appear on the corrupted data channels, are presumably best represented as a subspace of the corrupted data channels themselves rather than a subspace of the noise channels (i.e, artifacts in **X** are best represented by candidate noise components **U**, which were created from **X**).

Step 3

The projection from the noise components onto the channels is calculated. We recommend using a least squares solution to solve the regression problem (e.g., using matrix division in MATLAB).

$\mathbf{X}_{MC}$ = **X** - *mean*(**X**)	(4)
**ProjectionMatrix** = *mldivide*(**BadCompActivity** , $\mathbf{X}_{MC}$)	(5)
**ProjectedNoise** = **BadCompActivity** * **ProjectionMatrix**	(6)

Commentary: An alternative approach to calculate the projection is to apply a Moore-Penrose pseudoinverse to the **A** and/or **B** unmixing matrices, but we have found that it does not perform as well. The projection calculation, just like the CCA calculation, can be done on the same window of the data the user wants to clean, or from another segment of data. If the relationship between the EEG and noise sensors is reliable/repeatable across time, the steps of identifying the noise subspaces and/or projections could be performed once on a separate set of data. Thus, real-time implementation could be assisted by removing unnecessary computation.

Step 4

The projected noise is subtracted from the corrupted data channels.

$\mathbf{X}_{Clean}$ = **X** - **ProjectedNoise**	(7)

Commentary: Because the noise components are calculated as linear mixtures of the original recordings (**X** and/or **Y**) and because the projection onto the channels is also linear, iCanClean noise cancellation resembles a spatial filter. Note that if employed in a moving window, iCanClean's equivalent spatial filter could be time-varying rather than fixed, which should be better able to handle non-stationary data.
