# Supplementary figures and images for: iCanClean Removes Motion, Muscle, Eye, and Line-Noise Artifacts from Phantom EEG
Source: Sensors (Basel). 2023 Oct 1;23(19):8214. doi: 10.3390/s23198214 (PMC10574843; doi:10.3390/s23198214)

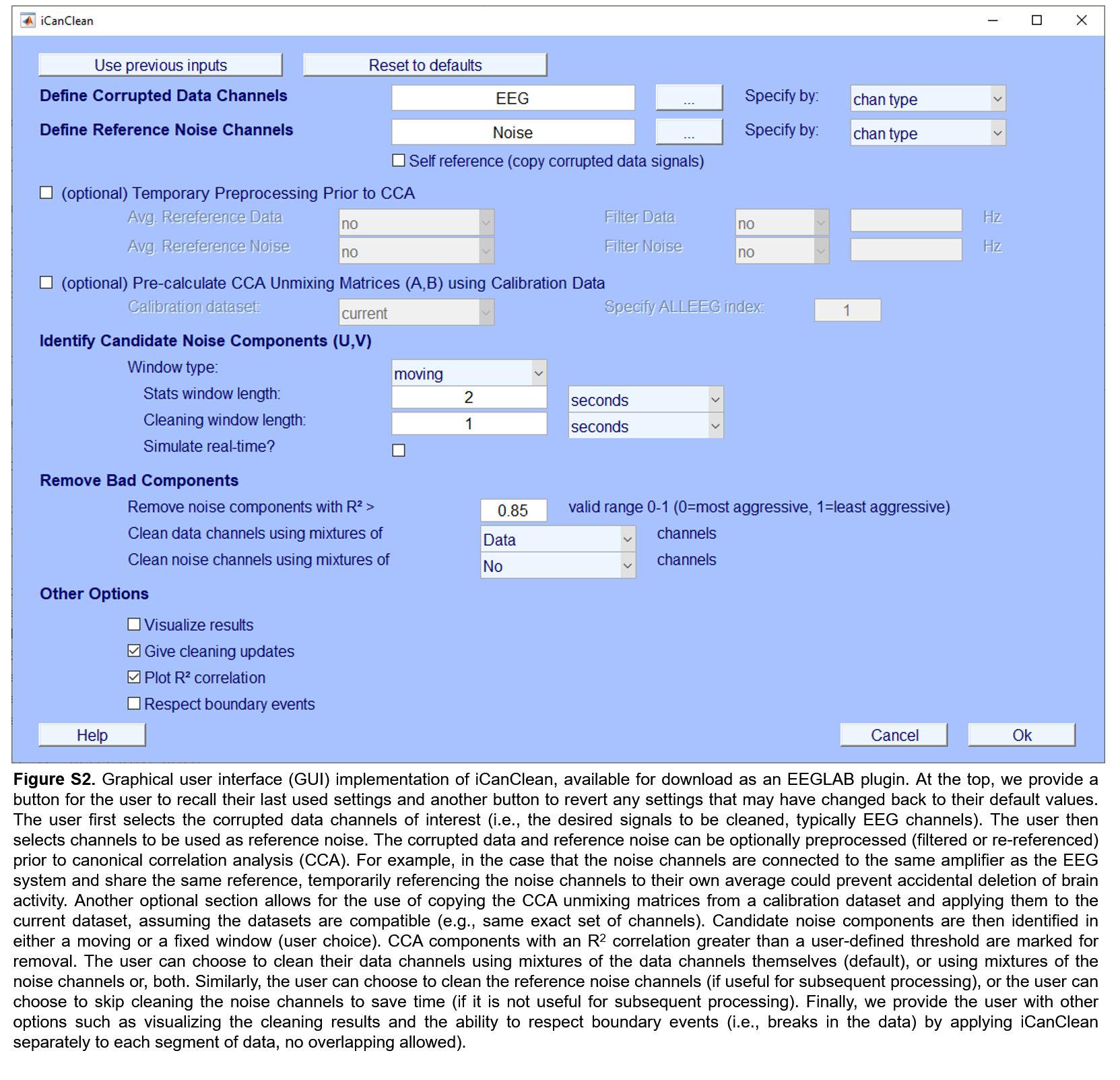

Supplement: Supplementary file 1 [file sensors-23-08214-s001.zip › Figure S2.png]

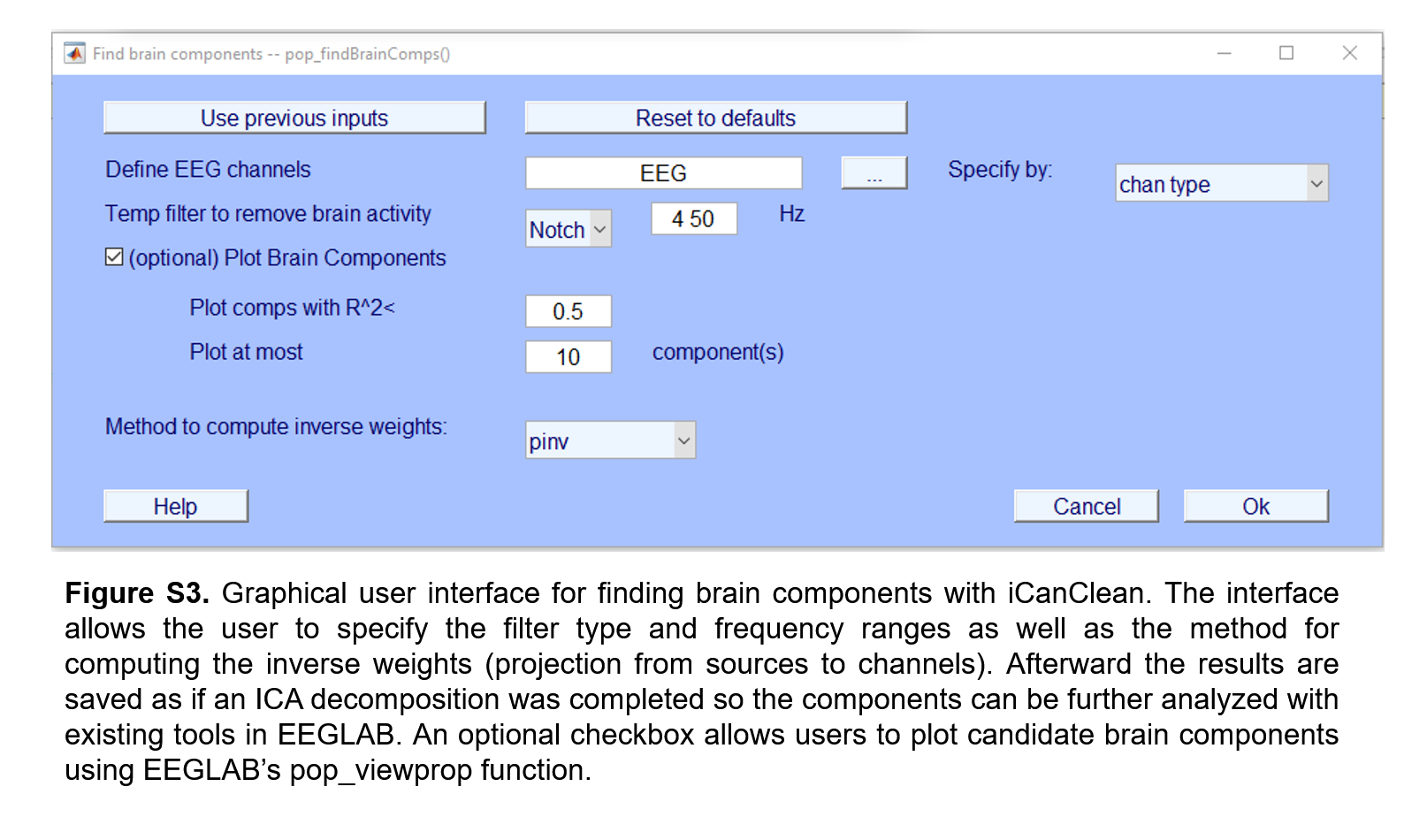

Supplement: Supplementary file 1 [file sensors-23-08214-s001.zip › Figure S3.png]

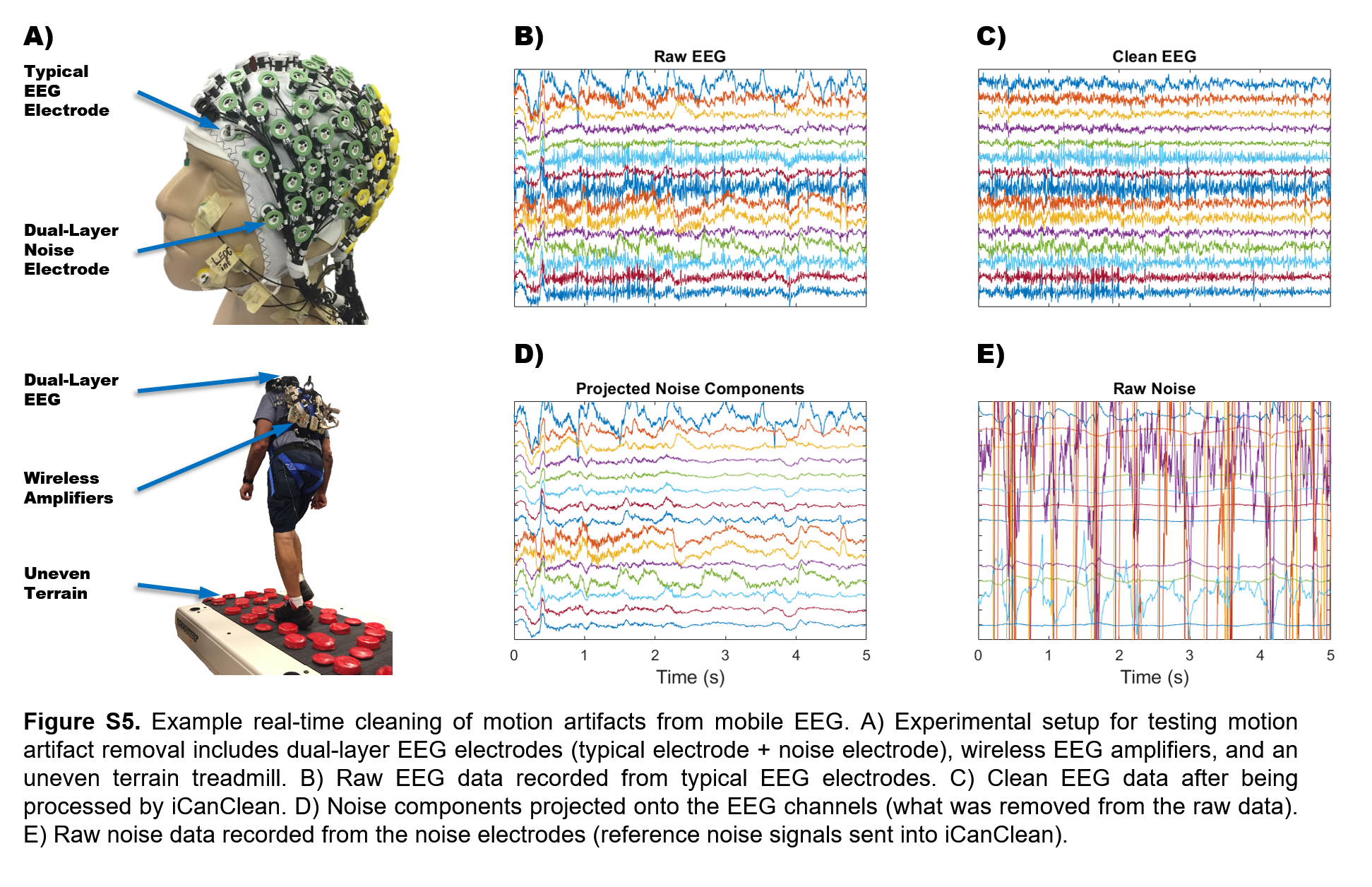

Supplement: Supplementary file 1 [file sensors-23-08214-s001.zip › Figure S5.png]

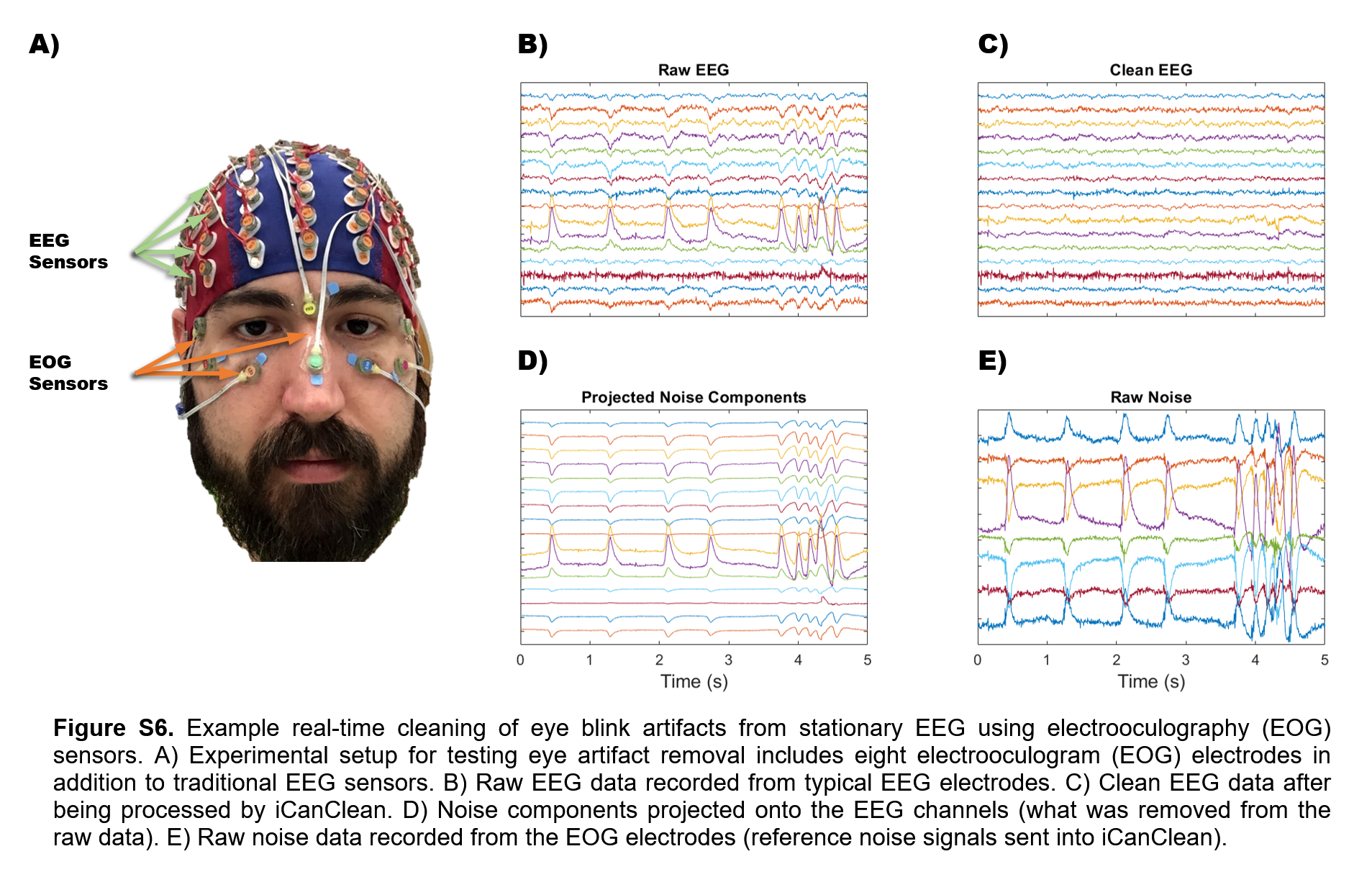

Supplement: Supplementary file 1 [file sensors-23-08214-s001.zip › Figure S6.png]
